# Enacting Smart Pedagogy in Higher Education Contexts: Sensemaking through Collaborative Biography

**DOI:** 10.1007/s10758-021-09495-5

**Published:** 2021-02-11

**Authors:** Vicente Reyes, Katherine McLay, Lauren Thomasse, Karen Olave-Encina, Arafeh Karimi, Mohammed Tareque Rahman, Lalanthi Seneviratne, Tran Le Nghi Tran

**Affiliations:** 1 School of Education, University of Nottingham, Nottingham, England; 2 School of Education, University of Queensland, St Lucia, QLD Australia; 3 Universidad de las Américas, Facultad de Educación, Viña del Mar, Chile; 4 School of Information Technology and Electrical Engineering, University of Queensland, St Lucia, QLD Australia; 5 Center for Excellence in Teaching and Learning, University of Liberal Arts Bangladesh, Dhaka, Bangladesh; 6 School of Education, University of Southern Queensland, Darling Heights, QLD Australia; 7 School of Languages and Culture, University of Queensland, QLD, Australia; 8 Banking Academy, Phu Yen Campus, Vietnam

**Keywords:** Smart pedagogy, Australia, Higher education, Collective biographies, ICT, Sensemaking

## Abstract

Scholars and practitioners argue that information and communication technology (ICT) provides flexibility of time and place and softens boundaries between students’ learning lives. The fluid movement between formal and informal learning contexts afforded by digital technology has prompted a re-definition of higher education learning environments to harness its potential. Further, technology can cater to diverse learners and promote lifelong learning in ways that the traditional didactic settings characteristic of tertiary contexts cannot. Scholars and practitioners have labelled this new teaching and learning landscape as smart pedagogy. This article engages with this scholarship by analysing a specific Australian case study in which ICT reforms have been deliberately implemented to adhere to smart pedagogies. Using collective biographies as a methodological tool, this inquiry provides insights into sensemaking experiences of a group of university academics whilst implementing ICT reforms anchored on Smart Pedagogy.

## Re-Structuring a Technology Course

This inquiry interrogates the essential role played by teacher educators (university lecturers) in attempting to implement ICT-intensive learning reforms that are anchored on smart pedagogy. Current literature on the interface of teaching and technology reveals that “limited research is available in a higher education context” (Rienties, Brouwer and Lygo-Baker 2013, p. 124). Moreover, scholarly work on smart pedagogies of teaching and learning reveal “fragmented studies on the didactic aspects of technology usage” (Daniela, & Lytras 2018, p. 2). The scant literature available on this important theme underscores the lack of “universal satisfaction” as regards “progress that has been made to integrate new technologies into teaching” (Kinchin 2012, p. E43). (Reyes et al.’s) critique of technological educational reforms in higher education contexts in Australia unearths “various stages of disconnect between ICT knowledge and ICT practice” (2018, p. 17). They further recommend that future research must necessarily be undertaken with a “nuanced approach” where existing concepts “needs to be problematized” (Reyes et al. 2017, p. 17). This article engages with these debates as it provides an exploration of teacher educators’ experiences in implementing ICT-intensive learning innovations in an Australian higher education context.

A viewpoint advocating for schools to be technology-rich environments that champion ‘twenty-first century learning” (Nielsen et al. 2015) has indubitably become embedded in Australia. This is represented primarily by the Teaching Teachers for the Future (TTF) initiative started almost a decade ago. TTF involved all 39 Australian teacher education institutions in a project funded by the Australian Government (Australian Government 2010). The aim was to build the capacity of Australian preservice teachers to equip school students with the requisite ICT knowledge and skills to engage productively in twenty-first century work and life. Our case study is a by-product of TTF.

The course under scrutiny is Digital Technologies in Contemporary Classrooms (DTCC^1^), a 13-week, undergraduate Education course that focuses on technology for teaching and learning for Pre-Service Teachers (PSTs). The course has run since 2011 and student feedback has reflected a fairly consistent level of dissatisfaction during that time. As part of a broader review of the School of Education in 2016, DTCC was identified as a course that needed reinvigoration. Two coordinators were appointed to reimagine and operationalise a new version of the course for implementation in 2017 and encouraged to experiment and take risks.

Previously, DTCC had been organised similarly to other courses in the undergraduate programme; that is, a one-hour lecture and a 1.5 h tutorial. In recent years, steps had been taken to make the course more engaging. For example, lectures deployed a flipped classroom model—prior to attending lectures, students were expected to watch a short learning video on related content. Additionally, the lectures had put active learning principles into practice: For example, using online real-time polls to glean student input, interspersing didactic instruction with discussion activities such as Think Pair Share and capturing student questions and comments during lectures using tools such as Padlet. Assessment in DTCC remained similar to other courses: attendance at tutorials was compulsory, formally recorded and comprised 10% of the total assessment. The other two assessment tasks were completed individually: a multi-modal oral presentation (30%); and a critically reflective blog (60%).

The reimagined 2017 course involved further reforms, targeting the remaining ‘traditional’ aspects of course structure and assessment by (1) having fewer face-to-face lectures (five in total) and replacing the remaining eight lectures with short learning videos; (2) redesigning assessment tasks so that three of the four were completed as group tasks with no individual results; and (3) organising tutorials as a three-week cycle: Sandpit-Synergy-Showcase. These design principles were clearly anchored on smart pedagogy emphasising the need for courses to be “developed collaboratively, by cross-disciplinary teams, finding consensus and negotiating environments through plurality and diversity” with the goal of attaining meaningful learning experiences” (Lorenzo and Gallon 2019, p. 44).

A key element of this cyclical approach involved tutors acting as critical friends and co-learners with students. Rather than didactically teaching students how to use various ICT tools, students instead get into the ‘sandpit’ to explore ICTs, that are freely available for use in future school setting together. With their tutor as a critical friend, students draw on one another to experiment, play, make mistakes, solve problems and plan the digital product they will cooperatively create. The following week (Synergy), each small group presents their work-in-progress in a relaxed environment, obtaining feedback from their tutor and peers to refine and improve their work. Finally, students formally present the final digital artefact they have cooperatively produced. Students are assessed on both their multimodal presentation and the digital artefact and a single result is given to each group.

## Reflecting on the Experience: Collective Biographies

Collective biography emerged in the 1980s from the work of Haug (1987) and became more widely accepted as a method in the 1990s (Wengraf et al. 2002), particularly among studies on school improvement (Butt and Raymond 1989). In collective biography, a group of researchers works together on a particular topic by drawing on their own memories. The shared work of telling, listening and writing helps transcend clichés and usual explanations, bringing written memories as close as possible to an embodied sense of ‘what happened’. In working this way, we do not conceive memory as ‘reliable’ in the sense of proving unquestionable facts.

Nor do we take what initially surfaces as being truer or more valid than the texts that are worked and reworked into the final written product. Rather, talking around our memories and listening to the detail of each other’s memories, are technologies that enable us to produce, through attention to the embodied sense of being in the remembered moment, a truth in relation to what cannot actually be recovered – the moment as it was lived. This is not a naïve, naturalistic truth, but a truth that is worked on through a technology of telling, listening and writing. In a sense, it is the very *unreliability* of memory that enables this close discursive work (Davies and Gannon 2006a, b, p. 3).

Apart from its ability to provide accounts that is deliberately constructed, collective biography also enables us to explore the ways in which “we resist and speak back to politics” that in most occasions permeate the learning and teaching environments (Charteris et al. 2017, p. 343).

The data for this collective biography were generated by the authors: academics who co-taught the revamped DTCC course in Semester 1, 2017 at the School of Education. Among the group, there are two course coordinators and seven tutors, each taking one or two tutorial groups of 15–21 students. Total enrolment was 450 and there were 16 tutorial groups. Students attended a 1.5 h tutorial each week. Tutorials followed a Sandpit–Synergy–Showcase cycle and drew on a flipped classroom model. Every three weeks, students worked with a different ICT tool for teaching—respectively Pinterest, Prezi and Powtoon. Tutors acted as critical friends who facilitated students in their exploratory journeys of using educational technologies for teaching and learning. A one-hour lecture was also timetabled each week for the course; however, only five ‘traditional’ face-to-face lectures were delivered. In the weeks when there were no lectures, students instead watched short learning videos produced by the course coordinators and tutors. Students were also encouraged to use the timetabled lecture hour to engage with peers in their small groups.

## Sensemaking: Writing, Telling, Analysing and Re-Storying

Sensemaking is a search for plausibility and coherence, that is reasonable and memorable, which embodies past experience and expectations and maintains the self while resonating with others. It can be constructed retrospectively yet used prospectively and captures thoughts and emotions. (Brown et al. 2008, p. 1038).

As a group, we initiated efforts towards sensemaking, searching “for plausibility and coherence” as we shared, reflected upon and interrogated what for most of us was a novel teaching and learning terrain: smart pedagogy. More specifically, we wanted to explore our teaching and learning practices with a “focus on sensemaking” particularly what we perceived was our joint experiences of “being thrown into an ongoing, unknowable, unpredictable streaming of experience in search of answers to the question” (Weick, Sutcliffe, & Obstfeld 2005, p. 410). We affirm and place great importance to what scholars and practitioners say in relation to putting greater “scholarly attention about how both instructors and students engage in sense-making strategies in technology-rich learning environments” (Fairchild, Meiners, and Violette 2016, p. 104).

Our sensemaking began way before the start of the DTCC course and picked up pace several months after the course was completed. Three months after the conclusion of the course, we each wrote a 500 word narrative detailing our personal experience as a tutor in DTCC. These narratives explored two main questions:

1. What are my personal experiences as a tutor in implementing a new course (DTCC) that attempted to incorporate technology into teaching?
2. How did I make sense of the challenges that I encountered in teaching DTCC?

The eight narratives were uploaded to a shared Dropbox folder, followed by discussions. In three, two-hour meetings, we read our narratives aloud and discussed and reflected on one another’s experiences. These conversations were very organic. No one was designated to lead the meeting and no particular structure or direction was imposed. Instead, we freely explored and shared our similar experiences as well as points of difference, asked questions and sought clarification. While the narratives varied in approach, content and style, some common themes became apparent “within the encounter, intra-action or the entanglement of agencies, the significance of matter, the movement from perception and affection to percept and affect and diffraction as concept and practice” (Davies and Gannon 2012, p. 357). To document the lively discussions, retain the moments as they went by and record the themes that emerged through our organic interactions, minutes were kept along with comments added to the Dropbox.

We recognised several recurring themes arising from discussion about our narratives. These “entwined re-storied experiences” (Charteris et al. 2017, p. 344) helped shape our representations of reality, interpretations and subjectivity as we critically reflected on our experiences in DTCC. We revised our narratives by incorporating new or more nuanced thoughts developed during our conversations. We created the moment in which the memory is told, as well as the remembered moments (Gonick 2015) of enacting smart pedagogy into our teaching and learning processes in DTCC. We uploaded our revised narratives to the shared Dropbox folder for further review, reflection and feedback.

This article presents the themes we identified during the development process of our collective biography. The themes fall into three areas in relation to our sensemaking attempts at navigating smart pedagogy: (1) First and foremost was our collective realisation of the need to embrace discomfort whilst enacting smart pedagogy. This first theme resonates with scholarly claims about how “(t)eachers are active participants in the use of the technology together with the students” and realise the need to accept “discomfort in their use” (Daniela 2019, p. 18). Secondly, a recognition of our agency amidst varying levels of discomfort. The third and final theme is an acknowledgment of emerging identities as an integral part of our sensemaking experiences within what we—as DTCC tutors—collectively perceived as a novel terrain of smart pedagogy. These three themes that have emerged parallel the dynamic critical reflection process described by Larrivee (2000).

## Discussion and Analysis: Themes from our Sensemaking Narratives

### Embracing Discomfort

We share three of our sensemaking narratives that are linked by a common thread: embracing discomfort. The first one is from the perspective of a practitioner with years of teaching and management experience who transitioned into the training of initial teacher education. The next comes from someone with a broad spectrum of involvement in different teaching contexts. The last one is from a former school administrator who critically reflects on difficulties that he encountered as tutor. These viewpoints serve as vivid and varied accounts of how academics make sense of discomfort intersecting with smart pedagogy.

#### The Myth of the Digital Native and the Need to Blur the Boundaries

Kate: As an English teacher and Head of English, I had—like most teachers—accepted without question that I was teaching a generation of ‘digital natives’ and worked diligently to incorporate technology meaningfully into the English programs for which I was responsible. My research interest in technology for teaching and learning thus developed from praxis, as I grappled with the tensions and challenges of working with ICT in my classroom. I had a gnawing sense that the issues surrounding technology for teaching and learning were more complex than questions of access.

My practitioner experience and curiosity evolved into doctoral studies in which I explored the identity issues that arise from young people’s relationship with technology and the ways learners take up and reject various identities and ideological positions in relation to technology. Since transitioning to the tertiary sector as an initial teacher educator, the importance of interrogating the ontological issues that arise through human–computer interaction has become increasingly clear to me for two key reasons. First, technology is rapidly and constantly changing. Many of the devices and software currently used will be obsolete by the time our students graduate. Thus, while epistemological knowledge and skills around technology are important, the capacity to adapt and transfer knowledge and skills to new contexts and tools requires us to explicitly attend to our preservice teachers’ ontological development. Non-cognitive skills like adaptability, resilience and curiosity are just as critical to engaging with ICTs as facility with various hardware and software.

In this course, I found I needed to engage constantly and explicitly with these ideas, as many of my students were anxious about using technology. These first-year students, fresh from secondary school, are the quintessential ‘digital natives’ but, consistent with scholarly voices arguing that the ‘digital native’ is a myth, there was little evidence of their intuitive grasp of technology. This leads me to the second reason that attending to ontological development is so important for our preservice teachers.

While most of our PSTs are ‘millennials’ or ‘digital natives’, their attitudes towards technology reflect ideological positions that are not generationally uniform. In course feedback, one student commented that, “A lot of people in the course disagree with too much technological integration in the classroom”. This reveals an attitude of resistance to educational technology from PSTs who are, overwhelmingly, members of the ‘Net generation’ who have ‘grown up digital’. This challenges the widely-accepted view that today’s young people ‘need’ technology to learn and have intuitive technological skills. Further, our preservice teachers are not ‘blank slates’, but bring their own experience of and beliefs about schooling to their tertiary studies and to the profession. In short, preservice teachers already possess not only a range of knowledge and skills in relation to technology, but also varying values, attitudes and beliefs about technology in education.

#### Teaching Learning Versus Teaching Technology—Our Dilemma

Lauren: Throughout my classroom teaching experience, I have been privileged to observe dramatic changes in the technological landscape. These changes range from encountering an interactive whiteboard in London, to teaching via video correspondence in South Korea while based in Brisbane, Australia. In the not-too distant past, it would have been inconceivable to teach real-time lessons in another country! But perhaps my most profound encounter with technology’s potential to positively impact teaching and learning came from my teaching children with Autism Spectrum Disorder. These children, who so often fall between the gaps in inflexible mainstream classrooms, accessed learning opportunities through technology that would otherwise have been unavailable. This experience in particular has helped reframe my conception of technology. Instead of anxiety about technological challenges and failure, I have learned to welcome the learning and teaching opportunities that technology offers. Modelling and making visible this mindset for students in DTCC was critical to my practice and to my students’ success—they don’t just need to know ‘how’ to use this or that digital tool, they need to have the courage to try and fail, take risks, experiment, play and problem-solve when things don’t go to plan.

While most of my students are members of the ‘Net generation’, it was clear to me from the outset that many experienced discomfort and were reticent about trying new technology. To my astonishment, few students had used the tools with which we asked them to engage and if they had, many could not conceive of Pinterest, for instance, as an educational tool: “How could I possibly use this when I teach?” was a common cry. A key part of my role in tutorials involved moving from group to group and problem-solving with students, encouraging them to play with the new technology and to persevere, despite their obvious discomfort and frustration. The only way through discomfort and frustration is through it and, once the task was complete, many students found they could now see new ways of incorporating technology into the classroom.

While there were many wonderful ‘a-ha’ moments, I remain surprised by the number of DTCC students who, despite being ‘digital natives’, vowed that they would not incorporate technology into their classroom. Over time and with dialogue, the main reason for not embracing technology emerged – quite simply, students did not perceive themselves as technologically adept. Fear of failure outweighed curiosity and saw them turn away from the unknown. Explicitly engaging with Carol Dweck’s work on Growth Mindset (2006) was an excellent resource and helped me reframe student fears and reassure them that learning isn’t easy! Rather than a sign of failure, discomfort is a marker of growth and development. To me, this is the essence of learning; the meta-skill applicable to all learning experiences, not just those involving technology.

#### A shadow of a Real Teacher

Tareque: As a former school administrator with cross-cultural experience and extensive training in educational policy, one of the dilemmas that I grappled with was whether or not DTCC’s core idea, Technology, Pedagogy and Content Knowledge or TPACK and its framework really helps in teaching technology. This was grounded on lingering questions I had. I questioned myself all the time regarding students’ preparedness for collaborating. Are we taking the idea of digital natives for granted and therefore accepting that all these students would be familiar with ICT and be ready to engage with content and pedagogy? Shifting from a researcher who wants to understand students’ experience as they confront various educational policies and then again becoming a tutor who needs to evaluate students’ efforts was a continuing challenge.

As a tutor, I encountered resistance from students, particularly in their anxiety in using unfamiliar teaching tools. Being the tutor, I also experienced personal conflicts pertaining to one’s sense of empathy—knowing that the students struggled and persevered created internal conflict when marking an artefact that doesn’t capture students’ interior learning journey. Also, there was a tension between the requirements that we ‘mark’ a student on their output while also encouraging students to develop risk-taking dispositions (which might not pay off in terms of their grade/s).

Some students challenged the concept of using ICT for creating better learning environments as they believed that while ICT supplements pedagogy and content, in reality it is not easy to balance as most of the time ICT takes “all the lights of the show”. While incorporating ICT, in many cases I have experienced that it takes up a significant amount of student–teacher interaction time. Things that previously I was explaining to them with all my personal charisma suddenly became a lot more dependent on the technological tools which in some cases made me feel a shadow of a real teacher rather than the teacher himself.

Kate asserts that notwithstanding the discomforts of teaching and learning there is a need to address ontological aspects of learners whilst engaging in smart pedagogy. Lauren argues that discomfort should be seen as a marker of growth and identity. Tareque identifies the careful balance that he—as tutor—tries to manage in promoting risk-taking and creativity amongst learners engaged in smart pedagogy alongside the requirement of evaluating learners’ outcomes. In our sensemaking, we acknowledge the “ever‐changing configuration of interpretations that individuals attach to themselves” (Geijsel and Meijers 2006, p. 473) especially in the process of encountering discomfiting encounters.

### Agency Amidst Discomfort

We are informed by what scholars have claimed as a necessary by-product of human computer interaction. In our attempt at making sense of the discomfort that we encounter, we are aware that the actions we take in our interactions with technology “is endowed with a whole host of possible variables that can alter the agentic experience dramatically” (Limerick, Coyle, and James 2014, p. 1). We share another three of our sensemaking experiences, this time emerging from a realisation of how we carve out a sense of agency whilst embracing discomfort. The first one is from the perspective of an experienced English as a Second Language (ESL) teacher who comes to grips with the challenges of smart pedagogy in order to derive positive outcomes for learners. The second is from a practitioner well-versed with technology who is able to leverage on her skills and expertise to design approaches to enable learners to engage much more critically with technology. The third is from another seasoned ESL teacher who reflects on how she—as a tutor—taps on her funds of knowledge (Moll et al. 1992) in order to exercise her sense of agency.

#### Building a Bridge Across the ‘Deep End

Tran: When I trained to be an English teacher 20 years ago in Vietnam, ICT was not a significant part of the educational landscape. There was little emphasis on ICT in curriculum documents, few classrooms were equipped with a computer and internet connection was rare both in schools and homes. However, when I became a lecturer in 2007, I encountered a wide range of educational technologies available for both teachers and students.

As a new teacher and an old learner (in comparison to my then-students), I learned through trial and error—I made many mistakes as I struggled to support and enhance my teaching with ICT. I tried different pedagogical approaches, experimented with different technologies and learned from my students who were, in many cases, more technologically competent than me. However, there were barriers: technology did not always work reliably, internet access was unstable and I encountered resistance from both students and colleagues. Despite these challenges, I felt that on balance, technology enhanced the teaching and learning experience and my interest in this field evolved into doctoral studies focused on mobile learning.

I brought all these experiences to teaching DTCC, but found that some of my attitudes and beliefs shaped by these experiences were challenged. For example, I saw myself as a ‘digital immigrant’ and my students—freshly graduated from Australian high schools—as ‘digital natives’. I didn’t expect so many of my students to be negative about and resistant to engaging technology. Instead of ‘digital natives’, I would describe many of my students as technophobes—who saw me as a technophile! I was surprised to learn that many of my DTCC teaching colleagues found the same thing. In short, I learned that while most of our preservice teachers are ‘millennials’ who have grown up in the digital age, this does not guarantee that they like or feel confident using technology. Indeed, many of my students expressed a sense of feeling ‘lost’ at the beginning of the course—some were intimidated by using previously unencountered technologies; others were dubious about the educational value of digital tools. These feelings of anxiety and uncertainty were exacerbated by DTCC being the first and only education course taken by many of our students. They were not only confronted with unfamiliar scholarly texts and curriculum documents, but also by unfamiliar technologies—and assessment tasks requiring them to integrate the two in an imagined future classroom context.

It is perhaps understandable that many PSTs felt they have been thrown into the deep end before they learned to swim. Instead of the relative familiarity and safety of didactic instruction, they had to climb into a digital ‘sandpit’. No one held their hands and told them what to do; they had to problem-solve together and build a bridge to connect what they already knew with what they needed to produce. For some, this was frustrating—and frightening. I became adept at discerning when they needed me to intervene and what kind of support to give: critical feedback for improvement, affirmation, encouragement, redirection, or help with solving a technical challenge. As my students learned from doing, I learned alongside (both with and from) them as a critical friend.

#### Walking the Talk in Smart Pedagogy

Arafeh: I've directed a global mobile learning project in underserved communities working with K-12 teachers and students in incorporating mobile technologies in different settings. This was an invaluable learning experience to practice incorporating technologies into different learning contexts in diverse educational settings in countries such as Argentina, India, Tanzania Palestine, Indonesia, South Korea, Malaysia, United States and Uganda. I worked in a larger scale 5 year project in Malaysia to incorporate cloud based virtual learning environment (VLE) in all public schools in the country.

Tutoring for the re-designed DDTC course was an invaluable experience in what is possible when PSTs have hands-on experience with attempting to incorporate technology into their lessons. The lesson-study style of the course was a refreshing approach, using the iterative cycle of sandpit-synergy-showcase as a way to involve learners in a deeper reflective practice. My personal focus was how this iterative reflective process can engage learners in a more independent, self-directed learning experience and potentially increases learners’ ownership and control of their technology adoptions. I used technology to enhance my tutoring by providing a more data enriched reflective process using Socrative, Google forms and Plickers technologies. I was familiar with the tools that were used in the course but the meta view of curation, collaboration and creation was an interesting perspective that assisted me in framing technologies for students as not just a static tool but as a tool that they can control and design their lessons with. I found the flipped concept that was integrated to the course a practical way to expose students to new pedagogical approaches in using technologies in their lessons. This helped me to walk the talk rather than just deliver theories of how technology can enhance the content delivery experience for learners. Another interesting aspect was working with the teaching team with very different technology adoption backgrounds. Interacting with other tutors and observing their adaptation of the Sandpit-Synergy-Showcase process gave me new ideas on other possible ways to enhance teaching by integrating feedback loops in the learning process.

#### Reflexive Practitioner: Repertoire of Epistemologies on feedback

Karen: I have consistently incorporated technology, from blended learning systems to electronic whiteboards, into teaching and learning episodes and course design during my 15 years as tertiary English as a Second Language (ESL) teacher and program coordinator in Chile. While I have now taught DTCC for several years, my most recent experience has defined my positionality in this course as a reflective practitioner.

All my students were ‘digital natives’ but my increasing critical awareness of assumptions about digital natives saw me consciously shift my thinking and move away from generalizing and stereotyping students in terms of skills and practices. Instead, I considered them as situated individuals whose practices are dynamic and continuously evolving. While students brought diverse repertoires of practices to the class, the course required students to build new practices. I approached teaching by eagerly gathering information about students’ familiarity with the technological tools involved in the course and worked to value them as individuals who brought something unique to the learning environment.

I drew heavily on my research background to critically reflect on my understanding and cultivate feedback practices that would positively shape my students’ practices and beliefs. DTCC has been a powerful, eye-opening experience in terms of being reflexive about my research. I was regularly struck by how powerful effective feedback experiences can be for learners and became aware of the many dilemmas and tensions that arise through receiving and providing feedback. In particular, the importance of providing students with several opportunities to receive and provide feedback from and to their peers to gain experience in and better understand the concept and purpose of feedback in learning. Having been immersed in the field of feedback for some time, my epistemologies of feedback clearly differed from my students’. I realised that my greatest challenge was to align my epistemologies with my learners’; this demanded ongoing dialogue and negotiation of epistemologies and expectations during tutorials. While some students quickly grasped and engaged with the theory and practice of meaningful feedback, others found it more challenging to appreciate its importance. Dialogue and negotiation can create a space for teachers and students to develop shared epistemologies and an appreciation for underpinning values, attitudes and beliefs.

These narratives resonate with the adaptation (short-term) and reframing (long-term) sensemaking responses theorised by Fairchild et al. (2016). Tran exercises agency by undertaking timely interventions, as a form of adaptation to the teaching and learning challenges she encounters in smart pedagogical contexts. Arafeh examines the innovative design features of DTCC and acknowledges a reframing of her perspective on directing PST’s learning trajectories. Karen undertakes a critical reflection of her role as tutor, with a specific focus on the powerful impact of feedback jointly experienced by teachers and learners in technology-rich contexts.

### Emerging Identities

A recurring theme unearthed in our collective biographies is that in enacting smart pedagogy we regularly encountered discomfort which in turn became useful as an integral “part of the teachers’ identity” (Daniela 2019, p. 18). In our conversations, we agreed to be open “to develop new ways of being learners and teachers in relation to technology” (McLay and Reyes 2019a, p. 24). We now share two more narratives that highlight how identities emerged in the intersection of discomfort and smart pedagogy. The first one is from a seasoned teacher whose introduction to technology can best be described as learning by doing. The second one comes from the overall coordinator of DTCC whose reflections provide paradoxical issues of identity-creation and formation in the context of smart pedagogy.

#### Making Peace with Competing Identities

Lalanthi: I first encountered ICT for teaching and learning a decade ago, but not as a teacher – there was very little engagement with digital tools at the school where I taught. It was only when my daughter started learning ICT as a subject in Year 4 that I ventured into the world of technology for teaching and learning. I tried to help my daughter, but I struggled with my lack of ICT knowledge and competence and I felt I would never be able to use technology effectively, much less teach with or about it! However, my transition to the tertiary sector in Sri Lanka compelled me to use ICT for teaching and learning.

I first had to learn how to use these ICTs myself—a source of some anxiety, even though there was only relatively limited access to ICTs in that context. Additionally, most of my students were from rural areas in Sri Lanka with low socio-economic backgrounds. Between students’ lack of exposure to technology and my own, it sometimes felt like the blind leading the blind! I have particularly vivid memories of working with a vision-impaired student for whom very specific software was provided to support his English language competency. In each of these experiences with technology, I found myself compelled to engage with ICT. Not unlike Tran’s students, I was thrown in the deep end but as my competence and confidence increased, I discovered many ways to use technology to support student learning.

I had to work hard to find interesting material and develop learner-centred practices to keep them engaged and active. I drew heavily on these experiences when teaching DTCC, where I encountered similar student attitudes towards educational technology. Virtually all DTCC students had mobile devices and used a range of technologies in their lives beyond formal educational settings, but many were anxious about using unfamiliar ICTs in assessment tasks. Indeed, some were quite vocally opposed to the idea of technology-rich classrooms. Over time, I made sense of these tensions by conceiving them as connected to the changes in teacher and learner identities, roles and relationships that the course structure of DTCC demanded.

As a DTCC tutor, my role shifted constantly and fluidly between being a learner of ICT and a teacher of ICT, both in and beyond the four walls of our classroom. Prior to tutorials, I was a learner as I prepared myself to teach with digital tools I had often not previously encountered. As I worked to embed these tools into learning activities, I gained insights into and empathy for my students’ anxieties and challenges, as well as a sense of satisfaction when things worked well. Dialogue with and input from my teaching colleagues was incredibly valuable to me during preparation. During tutorials, there were instances when a student or students were highly competent using a particular technological tool and again, I became a learner. My students could sometimes find more efficient ways of navigating technologies and more innovative approaches to embedding them into teaching and learning. For my students to benefit from others’ expertise, I had to consciously step out of my teacher identity and invite students to take up this identity instead. As I reflect on DTCC, I find myself wondering how we can help students ‘unknow’ so they can innovate and experiment and support the dynamic and constant shift between ‘schooled’ teacher-student relationships and identities that are the by-product of a democratised, twenty-first century knowledge society.

#### Identities in Smart Pedagogies Within Performative Cultures

Vicente: My research interests have mainly been in making sense of education reforms. From 2007 till 2013, I was affiliated with the National Institute of Education (Singapore) In the hyper-competitive Singaporean education context, not only was reform primarily driven by advancements in ICT, there was a very strong accountability push that demanded outcomes from university academics, school administrators, teachers and learners (i.e. education stakeholders). Thus, my research trajectory focused on exploring how stakeholders made sense of education reforms whilst managing powerful and oftentimes powerful discourses, a direction in line with Ball͛’s (2003) thesis of the terrors of performativity. Schools implementing ICT reforms had to regularly report on how often academic staff used technology in their teaching. There was widespread perception among higher education colleagues that academic promotion and the highly-coveted tenure were more accessible to academics who conformed to the national developmental goal of transforming their research into commercially viable technology applications to industry (Cheah 2016). As an academic in that context, I witnessed and experienced “coercive accountability” and in most occasions felt like an “audited subject,” more specifically, “a depersonalized unit of economic resource whose productivity and performance must constantly be measured and enhanced” (Shore and Susan 2003, p. 62).

From 2014 to 2016, I moved to an Australian higher education teaching environment where almost 85% of tertiary students were studying online. The two-year experience provided me with a nuanced perspective on the opportunities and challenges of teaching in so-called blended learning or $\overline{\text{e}}\text{-learning}$ environments. Together with other colleagues, I continue to teach and research about the pedagogical issues that arise in e-learning, particularly in higher education settings. The areas that I explore revolve around how teachers and learners located in the interface of technology and teaching negotiate the discourses of performativity and learnification (Biesta (2015) that have been amplified within ubiquitous ICT intensive teaching contexts.

As a co-coordinator attempting to enact smart pedagogy into the newly-revamped DTCC, I was keenly aware of Foucalt’s panoptic gaze (1975). The remit received in re-designing the course was to ensure high-student satisfaction. This clearly became what Foucalt describes as a disciplinary technology that guided my actions. Broadly, from my perspective I tried to make assessment, or in the particular case of DTCC, e-assessment for learning (e-afl) as the key lynchpin of the entire course. Lectures, tutorials and the sandpit-synergy-showcase model all revolved around e-afl. For me personally, the goals were to ensure that the students had $\overline{\text{a}}\text{genc}\underline{\text{y}}$ (initiate learning as active decision makers), in Biesta’s descriptions and that they could tap on to their $\overline{\text{f}}\text{unds \, of \, knowledg}\underline{\text{e}}$ for adult learners in the tradition of Moll et al. (1992). Despite all the efforts that the entire team poured into enacting smart pedagogy that we felt would be attractive to our learners, the student feedback on the course was extremely polarised: some really liked it while others despised it. At the cusp of rolling out the second wave of DTCC and building on the insights we gained, I received explicit reminders from the university that student satisfaction numbers need to be better. The strong audit culture, this time in an Australian Higher Education context, with the message that “what was important was to minimise the discomfort by reducing contentious readings and watering down substance to produce ‘thin’ pedagogies” (Blackmore 2009, p. 868) was almost deafening. I once again felt like an audited subject, but instead of the pressure to convert research into industry applications, this time the push came from the need to satisfy students seen as customers.

Lalanthi’s journey with technology and the corresponding identity shifts that she experienced is informative. While Vicente’s continuing identity crisis wedged in between different waves of performative cultures is a sobering reminder of current education contexts. We view these lingering challenges while enacting smart pedagogies as opportunities to take “a broader view of learning as developing knowledge and skills (epistemology) as well as selfhood or identity (ontology)” (McLay and Reyes 2019b, p. 289).

## Concluding Reflections

Teacher beliefs are self-generating and often unchallenged. Unless teachers develop the practice of critical reflection, they stay trapped in unexamined judgments, interpretations, assumptions and expectations. Approaching teaching as a reflective practitioner involves infusing personal beliefs and values into a professional identity, resulting in developing a deliberate code of conduct. (Larrivee 2000, p. 293).

In an era of uncertainty and constant technological change, what it means ‘to be’ a teacher necessarily shifts as well. This becomes highly relevant as we all face an unprecedented and uncertain Covid-19 global pandemic that has forced us to re-structure the delivery of education and the use of technology. The democratisation of knowledge and constant change have irreversibly challenged the traditional model of teacher-as-expert, student-as-novice. To use technology meaningfully and effectively for teaching and learning, we must be able to adapt to new technologies and contexts. And adaptability and flexibility are as much about our attitudes towards technology as they are about our technological knowledge and skills—we cannot adapt and innovate if we are afraid of failure, unwilling to continue learning, or unable to reconceive our role and identity as teachers in ways other than as ‘experts’.

Through our collective biographies, we critically reflected on our teacher identities in higher education contexts and realised that these had become fluid as we attempted to navigate the challenges of integrating technology and teaching. Tran recognised the shifts from teacher as expert to teacher as learner. Karen gained an appreciation of the need to match her epistemological expectations with that of her students. Lalanthi grappled with the challenge to “un-know” previous skills and knowledges. Arafeh became acutely aware of the need to “walk the talk”. And Tareque wondered about how his pedagogy has become a shadow of what he idealised as a teacher.

Karkazis et al. stated that “pedagogy can become significantly smarter exploiting the technological evolution in multiple aspects of different values to the different user roles” they further claim that smart pedagogy can lead to “the creation of experiences through virtual or mixed reality to increase learning efficiency” (Karkazis et al. 2019, p. 448). We contend that the enactment of smart pedagogy, needs to be anchored on practice. Along with acknowledging technological affordance, very real issues that impact teaching practice need our careful attention.

Tensions associated with teaching content through technology, rather than teaching about the technology itself, became apparent throughout this course. Cultivating self-directed learning, where the learner actively engages with the content and critically self-reflects on their practice to inform future learning, was central to DTCC. Most strikingly, the challenge of teaching through technology also presented the greatest opportunity to teach the content. When students expressed distress as they grappled with a new technology, We seized the opportunity to guide them to adopt self-directed learning strategies which support deep learning (Hattie 2008). Once students understood technology as a tool to support the development of knowledge, skills and attitudes, they could step out of their comfort zone and into the discomfort of learning. From this comes the greatest insight of teaching and learning with technology: technological unknowns arouse uncertainty, discomfort and vulnerability while simultaneously acting as a vehicle for development. As students made connections between theory and practice, technology was transformed from inhibitor to facilitator of rich learning experiences.

The tensions were reflected on Kate’s musings about the need to address the individuality of PSTs and the need to help them reflect on their ideological becoming. Lauren recognised the importance of non-cognitive skills as vitally important alongside the push to teach PSTs technologies to teach. Karen experienced both the highs and lows of dialogical feedback. Vicente worried about the balance of curriculum, against student satisfaction as a manifestation of the almost overpowering pressure of performativity.

One of the key insights that we derive from our collaborative conversations is the need to reimagine and redefine the concept of ‘classroom readiness’ to achieve a shared understanding that preparedness for twenty-first century classroom life is less about mastering content and skills and more about accepting and embracing that mastery is no longer possible in relation to technology. To be more specific, in contexts of smart pedagogies for example, we argue that embracing discomfort is essential. As teacher educators, modelling and cultivating such ‘ways of being’ as teachers is critical not only to our preservice teachers, but also to their future students, who will be shaped by their teachers’ approach to technology for teaching and learning.

## Footnotes

1 This a pseudonym.
